# Mechanosensitive Ion Channel Piezo1 Activated by Matrix Stiffness Regulates Oxidative Stress-Induced Senescence and Apoptosis in Human Intervertebral Disc Degeneration

**DOI:** 10.1155/2021/8884922

**Published:** 2021-02-10

**Authors:** Bingjin Wang, Wencan Ke, Kun Wang, Gaocai Li, Liang Ma, Saideng Lu, Qian Xiang, Zhiwei Liao, Rongjin Luo, Yu Song, Wenbin Hua, Xinghuo Wu, Yukun Zhang, Xianlin Zeng, Cao Yang

**Affiliations:** Department of Orthopaedics, Union Hospital, Tongji Medical College, Huazhong University of Science and Technology, Wuhan 430022, China

## Abstract

Mechanical stimulation plays a crucial part in the development of intervertebral disc degeneration (IDD). Extracellular matrix (ECM) stiffness, which is a crucial mechanical microenvironment of the nucleus pulposus (NP) tissue, contributes to the pathogenesis of IDD. The mechanosensitive ion channel Piezo1 mediates mechanical transduction. This study purposed to investigate the function of Piezo1 in human NP cells under ECM stiffness. The expression of Piezo1 and the ECM elasticity modulus increased in degenerative NP tissues. Stiff ECM activated the Piezo1 channel and increased intracellular Ca^2+^ levels. Moreover, the activation of Piezo1 increased intracellular reactive oxygen species (ROS) levels and the expression of GRP78 and CHOP, which contribute to oxidative stress and endoplasmic reticulum (ER) stress. Furthermore, stiff ECM aggravated oxidative stress-induced senescence and apoptosis in human NP cells. Piezo1 inhibition alleviated oxidative stress-induced senescence and apoptosis, caused by the increase in ECM stiffness. Finally, Piezo1 silencing ameliorated IDD in an in vivo rat model and decreased the elasticity modulus of rat NP tissues. In conclusion, we identified the mechanosensitive ion channel Piezo1 in human NP cells as a mechanical transduction mediator for stiff ECM stimulation. Our results provide novel insights into the mechanism of mechanical transduction in NP cells, with potential for treating IDD.

## 1. Introduction

Low back pain (LBP) caused by the intervertebral disc (IVD) degeneration (IDD) significantly influences the living quality in patients and causes a large financial burden. Weight-bearing and repeated bending are high-risk factors for IDD [1]. During repeated mechanical stimulation, the excessive mechanical load on nucleus pulposus (NP) cells, as keys to the development of degenerative disc diseases, exacerbates IDD progression [2, 3]. However, until recently, detailed pathogenesis and effective treatment of disc degeneration after excessive mechanical load are still not fully elucidated.

Mechanical loading is not only the physiological function of human NP tissues but also an important characteristic of the NP tissue mechanical microenvironment. Pressure and extracellular matrix (ECM) stiffness are the main mechanical microenvironment in the NP tissue. With changes in the body position and weight bearing, the pressure in the NP tissue can fluctuate greatly, which influences the balance of the mechanical microenvironment of the NP tissue [1]. Compression stress can result in the accelerated functional transition of NP cells and ECM remodeling during the progression of IVD [4].

Among mechanical stimulations, matrix stiffness can profoundly control cell behavior, including proliferation, apoptosis, and differentiation [5–7]. The mechanical properties of ECM in the NP tissue are closely related to IDD, and ECM stiffness increases with increasing age and grade of the degeneration [8]. Matrix stiffness is closely related to NP cell shape, which is crucial in the NP cell phenotype, and ECM stiffness (0.3 kPa) can regulate the inhibition of F-actin polymerization, cell shape, clustered morphology, and subsequent transcriptional inactivation, which is involved in maintaining the healthy NP cell phenotype [9]. Moreover, substrate stiffness can regulate growth, apoptosis, and ECM metabolism of the annulus fibrosus cells [7]. Although the imbalance of the mechanical microenvironment caused by ECM stiffness is the principal factor that accelerates IDD, the detailed mechanisms of mechanical transductions, which respond to the conversion of mechanical signals into chemical signals, remain unclear.

Previous studies have confirmed that oxidative stress can result in increased concentrations of reactive oxygen species (ROS) and lead to IDD [10, 11]. The increase of ROS has close related to cell senescence and apoptosis. Oxidative stress-induced senescence and apoptosis have been identified as major risk factors of IDD [12–15]. Mechanical compression can increase ROS generation and aggravate NP cell senescence [3]. However, the mechanosignaling pathways underlying mechanical transductions remain elusive.

The mechanosensitive Piezo1 channel is a Ca^2+^ ion channel that responds to mechanical stimulations [16–18]. Piezo1 mediates mechanical transduction in basic life functions, such as vascular development, blood pressure regulation, bone formation, and innate immunity [19–22]. Piezo1 can sense shear stress, mediate neuronal sensing of blood pressure, and play a crucial role in maintaining blood pressure homeostasis [19]. Piezo1 is required for bone formation and can sense and respond to changes in fluid shear stress and microgravity and alter bone formation by affecting bone mass and strength [21, 23]. Moreover, inhibiting Piezo1 allows oligodendrocyte progenitor cells to maintain the activity in stiff tissues caused by aging [24]. In NP cells, Piezo1 can sense abnormal mechanical stretch stress and is involved in regulating NLRP3 inflammasome assembly and NP cell apoptosis [25, 26]. Thus, Piezo1 may play important roles in IDD. The potential roles of Piezo1 activated by stiff ECM and the mechanism of mechanical transduction require further study.

In the present study, we aimed to observe the effect of IDD on ECM stiffness of NP tissues and expression of Piezo1 and provide novel insights into the underlying mechanism of mechanical transduction in NP cell for treating IDD.

## 2. Material and Methods

### 2.1. Patient Tissue Samples

Lumbar NP samples were obtained from 18 patients (ten men and eight women; age range: 21–65 years) with lumbar disc herniation and ten patients (four men and six women; age range: 15–25 years) with idiopathic scoliosis. The study protocol was approved by the Ethics Committee of Tongji Medical College, Huazhong University of Science and Technology (no. S214). Written informed consent was obtained from all patients.

### 2.2. ECM Stiffness

The degree of IDD was estimated before spinal surgery by three experienced spine surgeons using the Pfirrmann magnetic resonance imaging (MRI) grade system (grades I–V) [27]. NP specimens were collected from 18 patients with lumbar disc herniation (grades II–III, 11 patients; grades IV–V, seven patients) and ten patients with idiopathic scoliosis (grade I, six patients; grades II–III, four patients). An atomic force microscope (AFM) (INNOVA, Bruker Nano, Inc, USA) was used to test ECM stiffness of one 20 *μ*m-thick slice at the center of the human or rat disc. The elastic modulus was calculated as described in previous studies [28, 29].

### 2.3. Isolation and Culture of NP Cells

Three lumbar NP specimens from patients with idiopathic scoliosis were used for NP cell isolation, as described in a previous study [11]. NP cells were plated and cultured at 37°C and 5% CO_2_ in Dulbecco's Modified Eagle's Medium/F12 (Gibco, Grand Island, NY, USA) containing 15% fetal bovine serum (Gibco) and 1% penicillin/streptomycin (Invitrogen, Carlsbad, CA, USA). The passage cells were seeded into hydrogels bound to polystyrene plates (Matrigen, Brea, CA) with different stiffnesses (soft:1 kPa, stiff: 25 kPa) for subsequent experiments. The culture medium was changed every three days.

### 2.4. Immunohistochemistry

Human NP specimens were fixed with formaldehyde, embedded in paraffin, and sliced into 4 *μ*m sections. Subsequently, the sections were incubated with the antibody against Piezo1 (No. NBP1-78537, Novus, Littleton, CO, USA). The Dako REAL™ En-Vision™ Detection System, Peroxidase/DAB+, Rabbit/Mouse (Dako Cytomation, Glostrup, Denmark), was used to stain the sections according to the manufacturer's instructions. The sections were imaged and analyzed via microscopy (Olympus, Tokyo, Japan).

### 2.5. Quantitative Reverse Transcription PCR (qRT-PCR)

Total RNA from human NP cells in different groups was extracted using TRIzol reagent (Invitrogen, Carlsbad, CA, USA). qRT-PCR was performed according to the manufacturer's instructions. PrimeScript™ 1st RT Master Mix (TaKaRa Biotechnology, Otsu, Japan) was used to synthesize cDNA from total RNA. qRT-PCR was performed using SYBR PrimeScript RT-PCR Kit (TaKaRa Biotechnology, Otsu, Japan) on the CFX connect™ Real-time system (Bio-Rad, USA). The primers of Piezo1 used for qRT-PCR were forward, 5′-ACTTTCTGGTGACCCTGCAC-3′, reverse, 5′-GGCAGGTACAGCCACTTGAT-3′. The relative RNA expression levels were normalized to GAPDH (forward, 5′-TCAAGAAGGTGGTGAAGCAGG-3′, reverse, 5′-TCAAAGGTGGAGGAGTGGGT-3′). The test was performed in triple replication. The relative Piezo1 expression level was analyzed using the 2^−*ΔΔ*Ct^ method.

### 2.6. Western Blotting Analysis

Total protein was extracted from human NP cells using radioimmunoprecipitation assay (RIPA) (Beyotime, Shanghai, China) buffer with 1 mmol/L phenylmethanesulfonyl fluoride (PMSF), and total protein concentrations were measured using BCA protein assay kit (Beyotime, Shanghai, China) according to the manufacturer's instructions. The proteins were denatured by heat and stored at -80°C if necessary. Proteins (30 *μ*g) from each sample were separated using surePAGE™ prefabricated gels (4-20%, Genscript, Nanjing, China) and transferred onto polyvinylidene fluoride (PVDF) membrane (Bio-Rad, Hercules, CA, USA). The membranes were blocked with 5% skimmed milk in Tris-HCl buffer saline containing 0.1% Tween-20 (TBST) and incubated overnight at 4°C with primary antibodies, including Piezo1 (No. NBP1-78537, Novus), GRP78 (No. 11587-1-AP, Proteintech, IL, USA), C/EBP homologous protein (CHOP) (No. 15204-1-AP, Proteintech), cleaved caspase-3 (No. 9664; Cell Signaling Technology, Danvers, MA, USA), Bax (No. 50599-2-Ig, Proteintech), Bcl-2 (No. 12789-1-AP, Proteintech), p53 (No. ab26, Abcam, Cambridge, UK), and p16 (No.ab51243, Abcam), and GAPDH (No. 10494-1-AP, Proteintech) was used as the internal reference protein. After incubation with appropriate HRP-conjugated anti-rabbit or anti-mouse secondary antibodies (Proteintech) (room temperature, 1 h), the protein bands were visualized using an enhanced chemiluminescence reagents (Thermo Fisher Scientific, Waltham, MA, USA) and detected using the ChemiDoc-It 610 imaging system (UVP, upland, CA, USA).

### 2.7. RNA Interference

Piezo1 silencing was achieved via Piezo1-targeting siRNA (Piezo1: stB0009164A) and the corresponding negative controls (RiboBio, Guangzhou, China). Cell transfections were performed using Lipofectamine 2000 (Invitrogen), according to the manufacturer's protocol.

### 2.8. Determining Calcium Levels

Intracellular Ca^2+^ levels were detected using the specific Ca^2+^-sensitive fluorescent indicator Fura-4-AM (MedChemExpress, Monmouth Junction, NJ, USA) according to the manufacturer's instructions. Briefly, after the cells were cultured and treated with stimulation or corresponding reagent, NP cells were incubated with 5 *μ*M Fura-4-AM for 30 min at 37°C in the dark, and intracellular Ca^2+^ levels were calculated by analyzing fluorescence images collected via fluorescence microscopy (Olympus).

### 2.9. Flow Cytometry

The apoptosis rate in human NP cells was assessed using an Annexin V-FITC/PI Apoptosis Detection Kit (Yeasen Biotech, Shanghai, China) following the manufacturer's protocols. Fluorescence emission peaks were analyzed using a flow cytometer (BD FACSCalibur; BD Biosciences, San Jose, USA).

### 2.10. ROS Measurement

Intracellular total ROS levels were measured using 2′,7′-dichlorofluorescin diacetate (DCFH-DA, S0033; Beyotime, Shanghai, China) according to the manufacturer's instructions. Subsequently, fluorescence emission peaks were detected using BD FACSCalibur (BD Biosciences).

### 2.11. Senescence-Associated *β*-Galactosidase (SA-*β*-Gal) Staining

SA-*β*-gal staining was performed using the SA-*β*-gal staining kit (Beyotime, Shanghai, China) following the manufacturer's instructions. Briefly, human NP cells were cultured in polystyrene plates with different stiffnesses (soft: 1 kPa, stiff: 25 kPa) for 24 h. Cells were washed with phosphate buffer saline (PBS) and fixed with 4% paraformaldehyde (15 min, room temperature), then incubated with fresh SA-*β*-gal staining solution at 37°C overnight (no CO_2_). The senescent NP cells with positive staining were imaged using a light microscope (Olympus). Five fields at least were selected for analyzation. The average percentage of SA-*β*-Gal-positive cells was analyzed.

### 2.12. Immunofluorescence

Immunofluorescence staining was implemented as described in a previous study [30]. Human NP cells in different groups were rinsed with PBS, fixed with 4% paraformaldehyde, and incubated with a primary antibody against Piezo1 (1 : 50, Novos) overnight at 4°C. After the cells were washed three times and incubated with a goat anti-mouse antibody (1 : 100; Abcam), nuclei were stained with DAPI (4,6-diamidino-2-phenylindole). The samples were imaged using a fluorescence microscope (Olympus).

### 2.13. The Rat IDD Model

Sprague–Dawley rats (three months old) were provided by the Laboratory Animal Center of Huazhong University of Science and Technology (Wuhan, China). All experimental protocols were approved by the Animal Experimentation Committee of Huazhong University of Science and Technology (No. S2394). The surgical procedure for constructing rat IDD models was previously described [11, 31]. The rat disc levels Co6/7, Co7/8, and Co8/9 were located, and the annulus fibrosus layer was punctured using a needle (27G) parallel to the end plates, after anesthesia with 2% (weight in volume) pentobarbital (40 mg/kg). Three groups, including Con+siNC, IDD + siNC, and IDD + siPiezo1, were prepared to evaluate the effect of Piezo1 on NP cells and IDD and the potential therapeutic effect of siRNA transfection in vivo. Precisely, 2 *μ*l of the siRNA solution was slowly injected into the target level, and each needle was kept in the disc for 10 s. Free unrestricted weight bearing activity of all animals was permitted.

### 2.14. Magnetic Resonance Imaging

Magnetic resonance imaging (MRI) of all rat tails was examined at 4 weeks after surgery and treatment. The T2 weighted images were obtained by using a BioSpec MRI (7.0 T/20 cm; Bruker, Billerica, MA, USA). According to the images, the Pfirrmann grades were used to evaluate the degenerative degree of the rat tails [27]. Subsequent immunohistochemical staining and histological assessment were performed on the discs after the magnetic resonance examination.

### 2.15. Immunohistochemical Staining and Histological Assessment in Animal Models

Rats were euthanized 4 weeks postsurgery. Rat IDD specimens were harvested, fixed with formaldehyde, decalcified, and embedded in paraffin. Subsequently, the specimens were sliced into 4 *μ*m sections. Then, the sections were stained with hematoxylin and eosin (HE), as well as Safranin-O (SO). The histopathological assessment was performed as previously described [32]. In addition, the immunohistochemical experiments were performed as described in previous studies [33]. Subsequently, the sections were incubated with primary antibodies against cleaved caspase-3 (Cell Signaling Technology) and p16 (Abcam) overnight at 4°C, and then the sections were incubated with appropriate secondary antibodies and counterstained with hematoxylin. The sections were imaged and analyzed via the digital pathology section scanning system (S360, Hamamatsu, Japan). The histologic grading scale includes the morphology (the shape and the constitution) and cellularity (the rate of stellar shaped cells and the location of proteoglycan matrix) of the NP tissue, the morphology (collagen lamellae and fibers) and cellularity (the proportion of fibroblasts and chondrocytes) of the anular fibrosus, and continuity of the endplates. The histological score of 5 is classified as normal intervertebral disc, 6 to 11 are classified as moderate IDD, and 12 to 14 are classified as severe IDD.

### 2.16. Statistical Analysis

Data are presented as the mean ± standard deviation (SD) of at least three independent experiments. Statistical analyses were performed using GraphPad Prism 8 software (La Jolla, CA, USA). The differences between groups were determined by Student's *t*-test or one-way ANOVA. *P* < 0.05 was considered statistically significant.

## 3. Results

### 3.1. Piezo1 Was Upregulated and ECM Stiffness Increased in Degenerative NP Tissues

**Figure 1:**
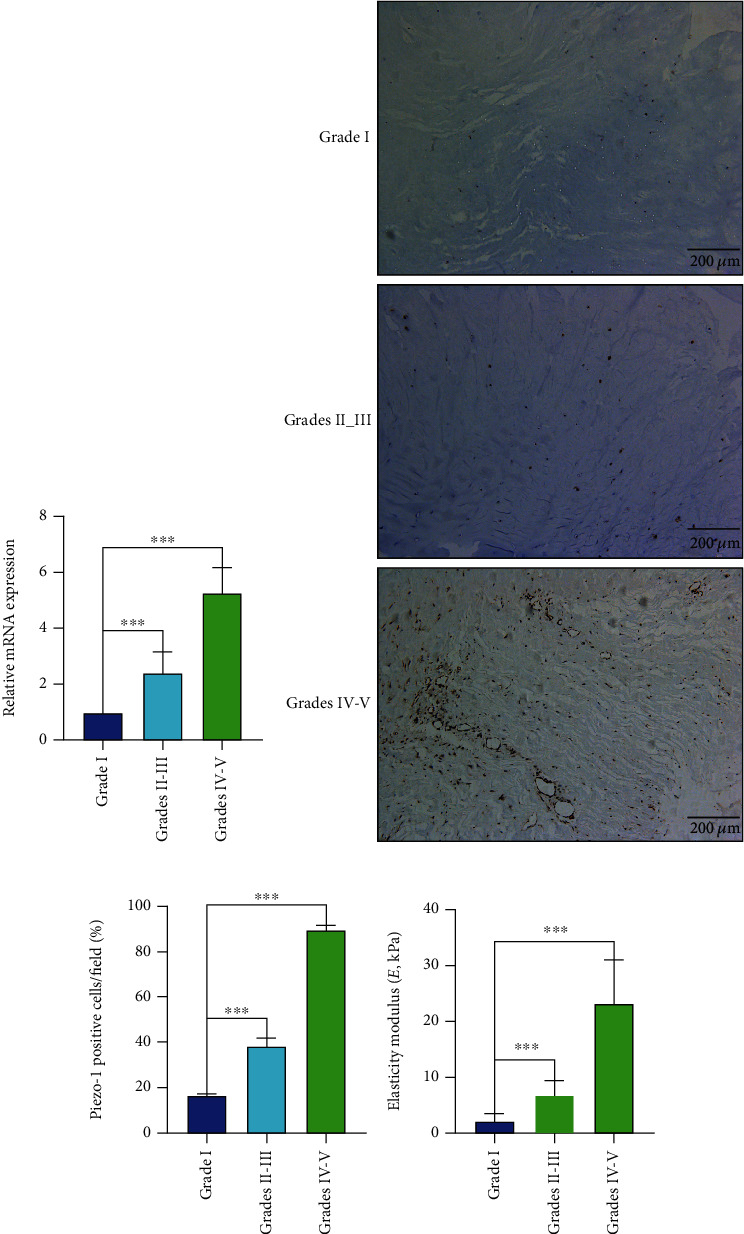
The Piezo1 expression and ECM stiffness in degenerative NP tissues. (a) The Piezo1 mRNA expression in human NP tissue specimens with different Pfirrmann grades was determined by qRT-PCR. (b) Immunohistochemistry analysis of Piezo1 in human NP tissue specimens with different Pfirrmann grades. (c) Piezo1-positive cells are presented as mean ± SD from three independent experiments. (d) The elasticity modulus of human NP tissue specimens with different Pfirrmann grades were measured via atomic force microscope (AFM) (^∗∗∗^*P* < 0.001, scale bar: 200 *μ*m).

The Piezo1 mRNA expression increased significantly in human NP tissue specimens with IDD contrasted to that in specimens with idiopathic scoliosis (Figure 1(a)). In addition, immunohistochemistry indicated that the percentage of Piezo1 positive cells increased in the grade IV-V group (Figures 1(b) and 1(c)). Furthermore, the elasticity modulus (~23 kPa) in the IDD group significantly increased, and the elasticity modulus in the grade I group was approximately 2 kPa (Figure 1(d)).

### 3.2. The Piezo1 Expression in NP Cells Was Upregulated in Response to Stiff ECM

**Figure 2:**
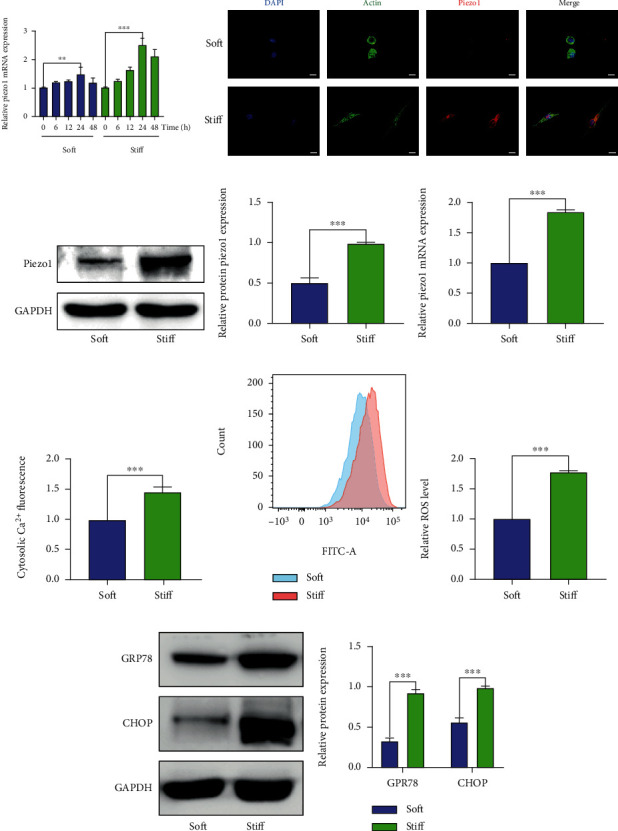
Stiff ECM induces the Piezo1 expression and activates oxidative stress and ER stress. (a) Piezo1 mRNA expression levels were analyzed by qRT-PCR in human NP cells cultured in polystyrene plates with different stiffnesses (soft: 1 kPa, stiff: 25 kPa) for 6, 12, 24, and 48 h. (b) The Piezo1 expression and cell morphology after 24 h of treatment were evaluated using immunofluorescence. Cytoskeleton was stained by phalloidin, and Cell nuclei are stained by DAPI. (c, d) The Piezo1 protein level was measured by western blotting. (e) Piezo1 mRNA expression levels were analyzed by qRT-PCR. (f) Intracellular Ca^2+^ levels measured using the specific Ca2 + -sensitive fluorescent indicator Fura-4-AM and analyzed via fluorescence microscopy. (g, h) The ROS levels were measured using DCFH-DA and analyzed using a flow cytometer. (i, j) GRP78 and CHOP protein levels were evaluated by western blotting. Data are presented as mean ± SD (^∗∗^*P* < 0.01, ^∗∗∗^*P* < 0.001, magnification: ×400, scale bar: 50 *μ*m).

According to the elasticity modulus measured in human NP tissues, NP cells were cultured in polystyrene plates with different stiffnesses (soft: 1 kPa, stiff: 25 kPa) for 6, 12, 24, and 48 h. The Piezo1 mRNA expression was assessed using qRT-PCR. As shown in Figure 2(a), there was a most significant difference in the Piezo1 expression after 24 h of treatment. Furthermore, immunofluorescence showed that the Piezo1 expression increased in stiff substrate (Figure 2(b)). NP cells on the soft substrate showed a round morphology, while elongated and spindle-shaped NP cells were present on the stiff substrate (Figure 2(b)). Additionally, western blotting, qRT-PCR, and corresponding qualification revealed that stiff substrate significantly increased the expression of Piezo1 at protein and mRNA levels (Figures 2(c)–2(e)). Stiff substrate not only increased the expression of Piezo1 but also activated the Piezo1 channel and increased intracellular Ca^2+^ levels (Figure 2(f)).

### 3.3. Stiff ECM Triggered the Increase of ROS and the Activation of Endoplasmic Reticulum Stress (ER) in Human NP Cells

To investigate whether stiff ECM triggered an increase in ROS and the activation of endoplasmic reticulum stress in human NP cells, the ROS levels in human NP cells were measured after treatment with different levels of stiffness. As shown in Figures 2(g) and 2(h), stiff substrate significantly increased the ROS level. Western blotting and relative quantitative analysis indicated that the protein levels of GPR78 and CHOP were increased in the stiff substrate group contrasted to those in the soft substrate group (Figures 2(i) and 2(j)).

### 3.4. Piezo1 Knockdown Attenuated Stiff ECM-Induced Increase in ROS and Activation of ER Stress

**Figure 3:**
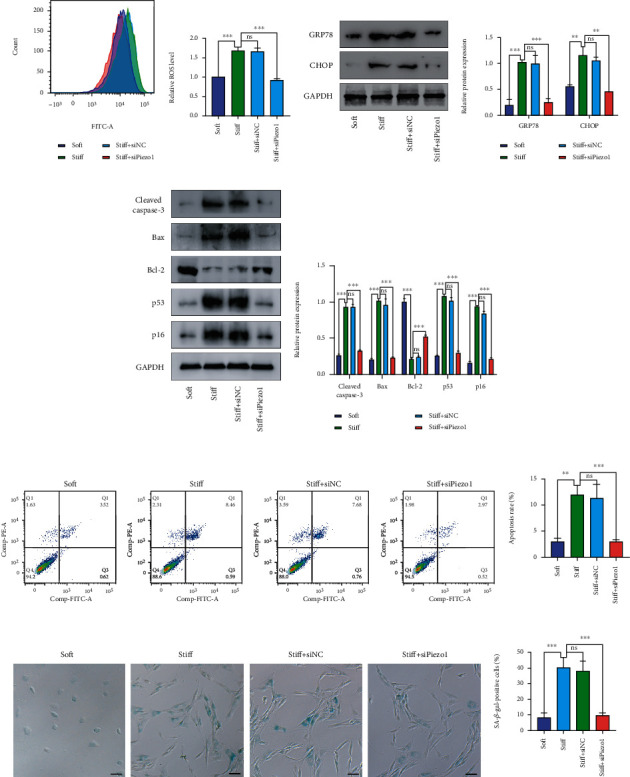
Piezo1 knockdown attenuates stiff ECM-induced oxidative stress and ER stress and oxidative stress-induced NP cell senescence and apoptosis. (a, b) The ROS levels were measured using DCFH-DA and analyzed using a flow cytometer. (c, d) GRP78 and CHOP protein levels were evaluated by western blotting. (e, f) The protein levels of cleaved caspase-3, Bax, Bcl-2, p53, and p16 were evaluated by western blotting. (g, h) Flow cytometry was performed to analyze the apoptosis rate in NP cells. (i, j) SA-*β*-gal staining of human NP cells and the average percentage of SA-*β*-Gal-positive cells in different groups, scale bar: 100 *μ*m. Data are presented as mean ± SD (ns: no significant; ^∗∗^*P* < 0.01, ^∗∗∗^*P* < 0.001).

The effect of Piezo1 on ROS levels and the activation of ER stress were verified via Piezo1 knockdown using siRNA. As shown in Figures 3(a) and 3(b), ROS levels increased in the stiff substrate group, but decreased significantly after Piezo1 was knocked down. These results showed that stiff substrates activated Piezo1 and exacerbated oxidative stress, and clarified Piezo1 plays an important role in ECM stiff-induced oxidative stress through Piezo1 silencing in human NP cells. Moreover, human NP cells transfected with siPiezo1 were treated with the stiff substrate. Western blotting and relative quantitative analysis indicated that GPR78 and CHOP expressions increased in the stiff substrate group. However, Piezo1 knockdown significantly decreased the expression of GPR78 and CHOP, which indicated that treatment with siPiezo1 attenuated stiff ECM-induced ER stress (Figures 3(c) and 3(d)).

### 3.5. Piezo1 Knockdown Attenuated NP Cell Senescence and Apoptosis Induced by Stiff ECM

The activation of oxidative stress can aggravate senescence and apoptosis in human NP cells. To investigate the effects of the stiff substrate, human NP cells were cultured under the stimulation of stiff ECM for 24 h. The results demonstrated that the expression of cleaved caspase-3, Bax, p53, and p16 increased, and that of Bcl-2 decreased significantly in the stiff substrate group (Figures 3(e) and 3(f)). As shown in Figures 3(i) and 3(j), compared with that in the soft substrate group, the average percentage of SA-*β*-Gal-positive cells increased in the stiff substrate group. In addition, flow cytometry showed that stiff substrate increased the apoptosis rate in human NP cells (Figures 3(g) and 3(h)). These results indicated that stiff substrates aggravated NP cell senescence and apoptosis. The effect of Piezo1 on stiff ECM-induced NP cell senescence and apoptosis was confirmed using siPiezo1. Pizeo1 knockdown in NP cells on stiff substrates downregulated the expression of cleaved caspase-3, Bax, p53, and p16 and upregulated that of Bcl-2 (Figures 3(e) and 3(f)). The results of SA-*β*-gal staining indicated that siPiezo1 significantly decreased the average percentage of SA-*β*-gal-positive cells (Figures 3(i) and 3(j)). Moreover, Piezo1 knockdown attenuated stiff substrate-induced apoptosis, manifested by a decrease in the apoptosis rate (Figures 3(g) and 3(h)).

### 3.6. siPiezo1 Partially Attenuated IDD Progression and Decreased ECM Stiffness In Vivo

**Figure 4:**
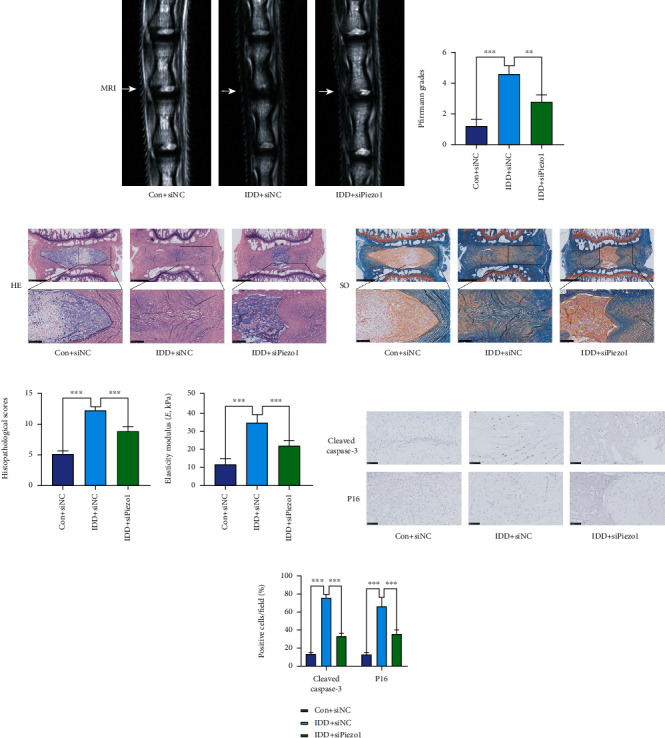
siPiezo1 partially ameliorates IDD and decreases ECM stiffness in vivo. (a) The representative T2 weighted MR images of rat tails after surgery and treatment. (b) Pfirrmann grades of different experimental groups at 4 weeks after surgery. (c) Representative HE staining images of disc specimens from different experimental groups, scale bar: 1 mm and 250 *μ*m. (d) Representative SO staining images of disc specimens from different experimental groups, scale bar: 1 mm and 250 *μ*m. (e) Histological scores in different experimental groups. (f) The elasticity modulus of rat NP tissue specimens from different experimental groups was measured via atomic force microscope (AFM). (g) Immunohistochemical experiments of apoptosis indicator (cleaved caspase-3) and senescence marker (P16). (h) Average percentages of cleaved caspase-3 and P16-positive cells in different groups, scale bar: 100 *μ*m. Data are presented as mean ± SD (^∗∗∗^*P* < 0.001).

To investigate the potential role of Piezo1 in IDD, IDD animal models were established using a disc puncture procedure in Sprague–Dawley rats. The representative T2 weighted MR images and Pfirrmann grades showed that the degeneration degree in the IDD + siNC group was severer than those in the Con+siNC group, the T2 weighted signal intensity was stronger than that in the IDD + siNC group, and the Pfirrmann scores in the IDD + siPiezo1 group were lower than those in the IDD + siNC group (Figures 4(a) and 4(b)). As shown in Figure 4(c), the NP tissue was oval-shaped and occupied a large volume of the whole disc in the Con+siNC group, detected using HE staining. In addition, high glycosaminoglycan content was confirmed in NP tissues as shown using SO staining in the Con+siNC group (Figure 4(d)). HE staining indicated the destruction of disc morphology, disruption of annulus fibrous, increased tissue fibrillation, and lower glycosaminoglycan content in the NP tissue in the IDD + siNC group (Figures 4(c) and 4(d)). Conversely, siPiezo1 ameliorated the changes in IDD compared to the IDD + siNC group, as clarified by the moderate boundary of the nucleus pulposus and annulus fibrosus and the disc height, although there was still a partial degenerative phenotype (Figures 4(c) and 4(d)). Moreover, histologic scores increased in the IDD + siNC group, but significantly decreased in the IDD + siPiezo1 group (Figure 4(e)). As shown in Figure 4(f), IDD increased the ECM elasticity modulus of the rat NP tissue, compared with that in the control group, and the ECM elasticity modulus in the IDD + siPiezo1 group decreased in vivo as expected. Moreover, immunohistochemical experiments of cleaved caspase-3 and P16 indicated that IDD increased the expression of apoptosis and senescence-related indicators ,and siPiezo1 attenuated NP cell apoptosis and senescence in vivo (Figures 4(g) and 4(h)).

## 4. Discussion

**Figure 5:**
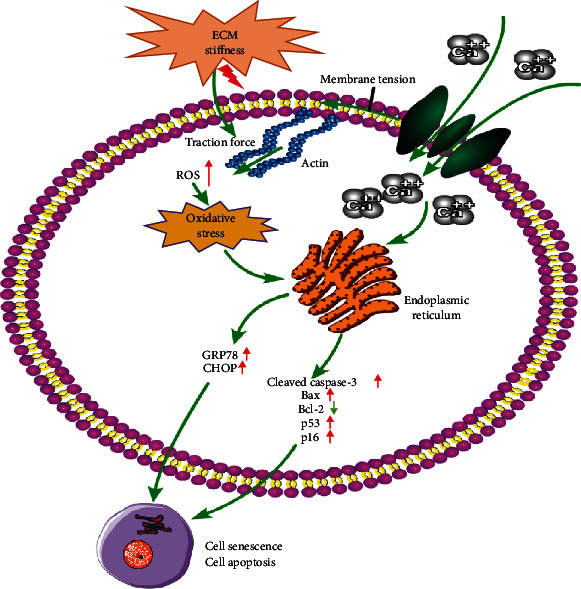
Schematic illustration of Piezo1 in human NP cells as a mechanical transduction mediator for stiff ECM stimulation. Piezo1, activated by matrix stiffness, increases intracellular Ca^2+^ levels and regulates ER stress and oxidative stress-induced apoptosis and senescence in human NP cells.

Piezo1, a mechanosensitive ion channel, responds several kinds of mechanical stimulations [21–24]. Extracellular mechanical stimulations are able to active the open of the Piezo1 channel and regulate the Ca^2+^ influx [16]. ECM stiffness, as one of the mechanical stimulations, may be a crucial regulator of NP cell phenotype, metabolism, and morphology [34]. However, the function of Piezo1 in human NP cells under the ECM stiffness stimulation is still unknown. In this study, the expression and function of Piezo1 in IDD progression were investigated. We demonstrated that the expression of Piezo1 and the ECM elasticity modulus increased in degenerative NP tissues. In addition, the results showed that Piezo1 activated due to matrix stiffness regulated oxidative stress-induced senescence and apoptosis in human IDD. Here, we identified the mechanosensitive ion channel Piezo1 in human NP cells as a mechanical transduction mediator for stiff ECM stimulation (Figure 5).

Piezo1 can be activated by mechanical stretch stimulation in NP cells, and the increase in intracellular Ca^2+^ levels involved in the activation of NLRP3 inflammasome [25]. The expression of Piezo1 is also upregulated under the stimulation of mechanical stretch stress or shear stress [23, 26]. Moreover, intracellular Ca^2+^ elevation disturbs intracellular Ca^2+^ homeostasis, which is closely related to ER stress [35, 36]. The ER stress leads to subsequent cell apoptosis. In chondrocytes, Piezo1, activated by mechanical stretch, plays an important role in ER stress-induced apoptosis [37]. Excessive ER stress can upregulate the expression of GRP78 and CHOP as ER stress markers [36, 38]. Moreover, previous studies have reported that excessive ROS can result in oxidative stress [39]. Similar with previous study, the elasticity modulus increased along with the increase of IDD degree in the present study [8]. The elasticity modulus in the NP tissue with Pfirrmann grades IV-V was about 23 kPa, so 25 kPa was selected as the stiffness of stiff substrate. The results indicated that Piezo1 was activated by stiff ECM, which resulted in intracellular Ca^2+^ elevation and increase in ROS levels, which activated ER stress and oxidative stress. The potential mechanism might be that the stiff ECM regulated NP cell morphology and changed the traction force in the cytomembrane. Previous studies had reported that extracellular mechanical stimuli regulated the traction forces, which activated the Piezo1 channel in the membrane subsequently [40]. In human NP cells, the activation of Piezo1 led to impaired intracellular Ca^2+^ homeostasis and increased ROS levels. Our previous study indicated that intracellular Ca^2+^ homeostasis impaired influenced the ER stress, and the intracellular ROS level had closely related to oxidative stress [36, 41]. Therefore, stiff ECM regulated ER stress and oxidative stress via the activation of the Piezo1 channel.

Oxidative stress involves in the regulation of apoptosis, senescence, ER stress, and autophagy [36, 41, 42]. Downregulation of Bcl-2, along with upregulation of Bax and cleaved caspase-3 in NP cells, is related to an increase of cell apoptosis [43, 44]. In the present study, stiff ECM decreased the expression of Bcl-2 and increased the expression of Bax and cleaved caspase-3, which indicated that stiff ECM increased the apoptosis of NP cells. Therefore, the apoptosis might be induced by the activation of ER stress and oxidative stress. The upregulation of p53 and p16 as senescence markers is common in IDD [45]. Mechanical stress can induce and promote rat NP cell senescence [46]. In our study, stiff ECM, as a mechanical stimulation, upregulated the expression of p53 and p16 in NP cells, which demonstrated that stiff ECM aggravated the senescence of NP cells. Accumulating studies have reported that excessive ROS accumulation is related to oxidative stress, and cellular senescence occurs under activation of oxidative stress; thus, excessive ROS accumulation contributes to cellular senescence [39, 47, 48]. In human NP cells, cellular senescence could be also due to excessive ROS accumulation and subsequent oxidative stress. Moreover, oxidative stress-induced apoptosis and senescence contribute to IDD progression [14]. The present results indicated that stiff ECM increases intracellular ROS, activates oxidative stress, and aggravates oxidative stress-induced apoptosis and senescence. The inhibition of Piezo1 attenuated oxidative stress-induced apoptosis and senescence, which indicated that stiff ECM increased oxidative stress-induced apoptosis and senescence via activation of Piezo1.

Piezo1 plays a crucial role in mechanical transduction, and its inhibition can reduce chondrocyte death after mechanical injury [49]. Piezo1 knockdown attenuates mechanical stretch stress-induced apoptosis in NP cells [26]. To the best of related knowledge, the relation between Piezo1 activated by ECM stiffness and oxidative stress or ER stress has not been investigated. In this study, Piezo1 inhibition decreased ROS levels and the expression of GRP78 and CHOP. The oxidative stress and ER stress activated by stiff ECM were inhibited by siPiezo1 in human NP cells. Moreover, Piezo1 knockdown alleviated oxidative stress-induced apoptosis and senescence. Finally, Piezo1 silencing ameliorated IDD in an in vivo rat model and decreased the elasticity modulus of rat NP tissues.

There are some limitations in the present study. First, similar with the animal models in previous studies [11, 31, 44, 50], a simple and effective rat acupuncture model is established to investigate the mechanism of mechanical stimulation in IDD and the potential therapeutic effects of Piezo1 inhibition. However, except for the stimulation of ECM stiffness, the acupuncture of rat tails also causes the biomechanical instability of intervertebral disc. The pathological mechanism of IDD may be related to interactive or associated effect of different biomechanical stimulations. The interactive relationship of different biomechanical stimulations in IDD should be investigated. Second, the mutation of the mechanosensitive cation channels Piezo2 may be responsible for symptoms of scoliosis. The NP tissues of Pfirrmann grade I were obtained from the patients with idiopathic scoliosis, but the potential genic changes were possible to influence our results about Piezo1. Further studies about the potential influences of Piezo on scoliosis should be conducted. Third, although the current work reveals the mechanism of IDD from a new perspective of biomechanics and oxidative stress according to the results in vitro and in vivo, the particular changing process of oxidative stress and ECM stiffness remains unclear. The histological evaluation in vivo at different time points is needed in further investigations.

## 5. Conclusion

The present results indicate that Piezo1 activation under stiff ECM is the mechanism underlying oxidative stress-induced apoptosis and senescence in IDD, leading to pathologic progression of IVD. Central to this mechanical signal transduction is the activation of Piezo1. We identified the mechanosensitive ion channel Piezo1 in human NP cells as a mechanical transduction mediator for stiff ECM stimulation. Our results provide novel insights into the mechanism of mechanical transduction in NP cells, with therapeutic potential for treating IDD.

## Data Availability

The data are included in the article to support the findings of this study.
